# Development of a hematogenous implant-related infection in a rat model

**DOI:** 10.1186/s12891-015-0699-7

**Published:** 2015-09-14

**Authors:** Stefanie M. Shiels, Katherine M. Bedigrew, Joseph C. Wenke

**Affiliations:** Extremity Trauma and Regenerative Medicine Task Area, US Army Institute of Surgical Research, JBSA-Fort Sam Houston, TX 78234 USA; Department of Orthopaedics, San Antonio Military Medical Center, JBSA-Fort Sam Houston, TX 78234 USA

## Abstract

**Background:**

Implant-related osteomyelitis is a major complication that requires immediate treatment, often involving removal of the implant, prolonging patient recovery and inflating expenses. Current research involving interventions to diminish the prevalence of such measures include investigating prophylactic and therapeutic remedies. A proper and accurate animal model is needed to thoroughly investigate such treatments. The scope of this project was to develop an animal model in which a consistent and measurable infection can be formed on an orthopedic implant when bacteria is introduced via a hematogenous source.

**Methods:**

Titanium Kirschner-wires were implanted into the intramedullary canals of both femurs. *Staphylococcus aureus*, ranging from10^4^ to 10^9^ colony forming units, was injected into a tail vessel. After a designated time (3, 7, 14, or 42 days) the femurs were harvested and bacterial numbers determined for both the femur and the implanted K-wire. In addition, histology and micro-computed tomography were used as subjective tools to further characterize the infection.

**Results:**

Consistent infection, that is infection of ≥75 % of the femurs, wasn’t achieved until 10^7^ CFU *S. aureus* was injected. At 10^7^ CFU, the femurs contained 4.6x10^6^ CFU/g bone tissue at day 3 and 4.8×10^8^ CFU/g bone tissue by day 14. The wire showed comparable contamination with 4.8×10^4^ CFU/mm^2^ at day 3 and 3.7×10^5^/mm^2^ by day 14. After 42 days, the bacteria number decreased but was still occupying at 1.9×10^5^ CFU/g bone tissue. There were morphological changes to the bone as well. At day 42, there were signs of osteonecrosis and active bone formation when compared to control animals that received a K-wire but were inoculated with saline.

**Conclusions:**

A model for hematogenous osteomyelitis, a common complication associated with implants, has been introduced. A reproducible, preclinical model is essential to evaluate future methods used to mitigate blood-borne bacteria hardware and bone infections.

## Background

Infections of implanted orthopedic devices can lead to implant failure, resulting in additional antibiotic therapies and possible surgical intervention, removal of the implant and subsequent revision of the implant site. Early onset colonization of an implant is often caused by introduction of bacteria into the surgical site during placement of the implant, however, another source of bacteria is the blood. A hematogenous infection, often from *Staphylococcus aureus,* originates from a secondary infection, most often from the skin, gums/teeth, or urinary tract, [1] and has traveled through the blood to the bone and implant.

Hematogenous osteomyelitis is a disease primarily found in children but is also associated with implants inserted in environmentally sterile conditions, such as those for total joint arthroplasties and fracture fixation of closed fractures. Hematogenous osteomyelitis in children is thought to be caused by the anatomy of the metaphyseal region of the long bone [2]. The vascular loop structure within growing long bones slows the blood flow through the region, localizing bacteria that may be in the blood. The disease presents as bacterial abscesses forming adjacent to the physis in the metaphyseal region of the bone, where bacteria can accumulate in the capillary beds (sinuses) [2]. Animal models for hematogenous osteomyelitis in children involve inoculation of bacteria, from a strain known to cause osteomyeltitis, into the bloodstream, often with the use of a sclerosing agent, such as sodium morrhuate, to entrap the bacteria in the blood vessels of the metaphysis of the bone and promote bacteria propagation [3–6].

Implanted medical devices, such as intramedullary nails or total joint prostheses, encourage bacterial colonization at the site of implantation and are therefore highly susceptible to infection [7]. Increased susceptibility to infection from *S. aureus* can be attributed to the mechanism of bacterial attachment to an implant and the deactivation of local granulocytes upon introduction of the implant itself [1]. The local immuno-incompetency creates an advantageous loci for infection [8]. Along with any bacterial contamination associated with surgery, there is a threat of infection throughout the life of the implant from hematogenous sources. Symptoms for implant-related osteomyelitis are similar to those experienced by children with hematogenous osteomyelitis, to include local pain and inflammation. In addition, implant-related infections may cause nonunion of fractures and prosthetic implant loosening. The implant serves as a nidus for bacterial attachment and biofilm formation, a collection of bacteria and matrix proteins that reduces the susceptibility of the bacteria to antimicrobials and the immune response, making implant-associated infections more difficult to treat.

Prevention of bacterial attachment and colonization to implants is a chief objective. Strategies include implant materials that reduce the ability of bacteria to colonize the implant and encourage interaction with surrounding tissue, regardless of when and how the bacteria comes into contact with the implant. Modifications to the implant could include coatings to decrease bacterial attachment or surface modifications to increase host-tissue integration [9]. Due to the low incidence of hematogenous osteomyelitis within patients, a reproducible animal model is needed to evaluate such interventions related to preventing orthopedic infections of hematogenous origin associated with prostheses and fixation devices. A model has been developed which will allow for screening of new materials and methods in a preclinical setting.

## Methods

### Bacteria preparation

A derivative of *Staphylococcus aureus* was used throughout the study. UAMS-1 (ATCC 49230), a well characterized methicillin susceptible osteomyelitis isolate, was modified to express green fluorescent protein [10]. *S. aureus* was streaked onto a sheep’s blood agar plate and incubated overnight at 37 °C. From the streaked plate, a select number of colonies were suspended in tryptic soy broth and incubated at 37 °C with agitation. When an OD_600_ of 0.2 was met, the bacteria was centrifuged at 4000 rpm for 10 min at 4 °C. A 12 % glycerol/TSB solution was added to the pellet and the bacteria were stored at −80 °C until use. Creating a stock of frozen bacteria limits the variance between surgery days. On the day of inoculation, a prepared frozen stock of UAMS-1 was thawed on ice and centrifuged at 4000 rpm for 10 min at 4 °C. The bacterial pellet was washed with normal saline and centrifuged again three times. After the last centrifugation, the sample was resuspended in normal saline at the bacterial concentration required for injection.

### In vivo surgical procedure

**Fig. 1:**
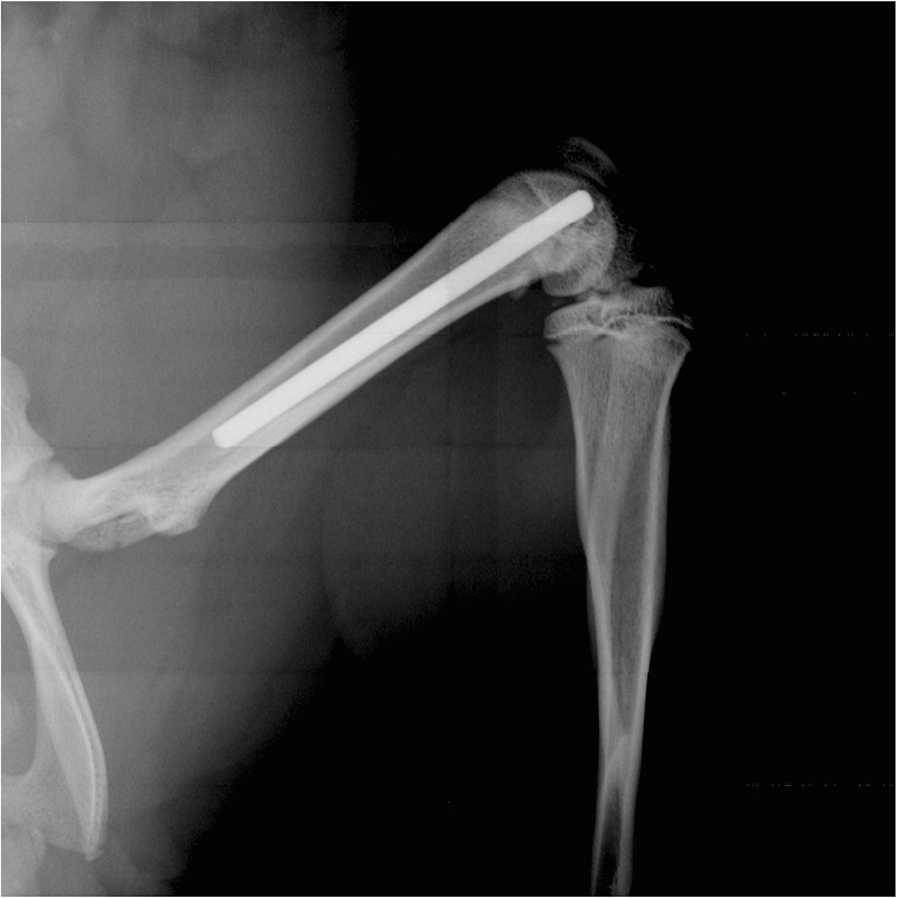
Surgical placement of a 1.25 mm diameter Ti alloy K-wire, 24 mm length

This study, approved by the Institutional Animal Care and Use Committee of the US Army Institute of Surgical Research, has been conducted in compliance with the Animal Welfare Act, the implementing Animal Welfare Regulations, and the principles of the Guide for the Care and Use of Laboratory Animals. Experimental design is described in Table 1. Sprague–Dawley Rats (male, 12.4 ± 0.14 weeks old, Harlan, Haslett, MI) received a smooth titanium Kirschner wires (K-wire; Ti-6Al-4 V, 24 mm length, 1.25 mm diameter; Synthes, Monument, CO) in the medullary canal of the femur, either bilaterally or unilaterally as described in the study design. Under sterile conditions, an incision was made over each knee and a medial parapatellar arthrotomy was created exposing the intracondylar notch. An 18-gauge cannulated needle was used to ream the medullary canal. A K-wire was inserted into the canal, unless otherwise stated, and the arthrotomy and skin incisions were closed. A high-resolution radiographic image (MX-20; Faxitron X-Ray Corporation, Tuscon, AZ) was taken of each operative limb to verify placement of the K-wire within the femoral canal (Fig. 1). Immediately following surgery, the animals were inoculated with 300 μL *S. aureus* in saline or saline alone via a tail vessel using a 24G catheter. The animals were recovered, individually housed, and offered water and food *ad libitum.* After the designated time period, the animals were weighed and humanely euthanized with FatalPlus®, and the femurs were aseptically harvested. To determine the bacterial bioburden on the wires, the K-wires were sonicated in saline and serial dilutions plated onto blood agar plates. To determine the bacterial bioburden within the bone, the bones were snap-frozen and crushed into a fine powder. A homogenate of the bone powder was made with sterile saline and serial dilutions of the saline was plated onto blood agar. The plates were incubated for 24 h at 37 °C. Bacterial colonies were enumerated and normalized to wire surface area or bone mass [11, 12].

**Table 1:** Experimental Design

	*S. aureus* CFUs	Survival (post inoculation)	n (femurs)/group/time point	Outcome measure
Experiment 1	0, 1×10^4^-1×10^9^	3-14 days	8	CFU bone, CFU wire
(bilateral)				
Experiment 2	1×10^7^	3-42 days	16	CFU bone, CFU wire, μCT, histology
(bilateral)				
Experiment 3	1×10^7^	3-42 days	6	CFU bone, CFU wire
(unilateral)

#### Experiment 1

The purpose of experiment 1 was to determine which bacterial inoculation concentration would produce a consistent and clinically relevant infection in the bone. Each animal received bilateral K-wires in the femoral canals. The animals were injected with a about 1×10^4^, 1×10^5^, 1×10^6^, 1×10^7^, 1×10^8^, or 1×10^9^ colony forming units (CFU) or saline, as a control. At 3, 7, or 14 days, the animals were euthanized and the limbs processed for enumeration of bacteria as described.

#### Experiment 2

The purpose of experiment 2 was to determine both the morphological change in the bone as well as the level of bioburden within the bone when the animals survived for 42 days. The longer survival time gave us the opportunity to characterize the chronicity of the infection and related changes to the bone structure. The animals received bilateral K-wires and about 1×10^7^ CFU of *S. aureus* or saline as a control via a tail vessel and were survived for 3, 7, 14, or 42 days to evaluate for a persistent infection. The bones were harvested, with up to four of the bones and wires per group placed into neutral buffered formalin before being scanned by micro-computed tomography (μCT) and processed for histology and the rest processed for enumeration as described.

#### Experiment 3

The purpose of experiment 3 was to determine the effect the K-wire had on establishing an infection of hematogenous origin within an operated femur. The animals received a unilateral K-wire and a sham surgery (up to include reaming) in the contralateral limb. The animals received an injection about 1x10^7^ CFU *S. aureus* via a tail vessel immediately following surgery. At 3, 7, 14, or 42 days, the animals were euthanized and the limbs and wires processed for enumeration as previously described.

For this study, the following criteria was used to define non-contaminated, contaminated, and infected bones and wires: values <10^2^ are below the limit of detection and are considered non-contaminated, values between 10^2^ and 10^3^ are contaminated, and values ≥ 10^3^ are infected.

### Micro-computed tomography (μCT)

After the wires were removed from the bone, the femurs were fixed in 10 % neutral buffered formalin. μCT images were acquired using a Scanco VivaCT40 (ScanCo, Bassersdorf, Switzerland). The femurs were scanned using a 21 μm voxel size at 55 kVp energy with a 120 s integration time. The images were converted to 8-bit bitmap files using Image J (NIH). Data Viewer (Bruker-MicroCT, Kontich, Belgium) was used to reorient the femurs along the longitudinal axis and recover images of the bones.

### Histology

After μCT, the femurs were decalcified with Shandon TBD-1 (ThermoFisher Scientific) before being dehydrated with increasing concentrations of ethanol and embedded in paraffin. Longitudinal sections (4 μm) were made through the reamed portion of the bone and stained with Hematoxylin & Eosin. The sections were examined using 4x and 20x Magnification for inflammatory cells, necrotic tissue, and bone remodeling.

### Statistical analysis

Multigroup comparisons were performed using a one-way ANOVA to compare bacterial bioburden at each time point followed by a Tukey’s post-hoc test for pairwise comparison. For experiment 3, a paired *T* test was used to compare bioburden, in the unilateral model, between the implanted and contralateral limbs. P values less than 0.05 were considered significant.

## Results

### Experiment 1

**Fig. 2:**
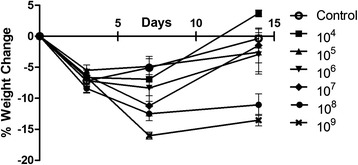
Weight Change of animals that received a bilateral K-wire with a hematogenous injection of *Staphylococcus aureus* or saline as a control. Group 10^7^, 10^8^, and 10^9^ were different from the control at day 7 with *p* < 0.05, 0.01, and 0.001, respectively. Groups 10^8^ and 10^9^ were different from control at day 14 with *p* < 0.001

**Fig. 3:**
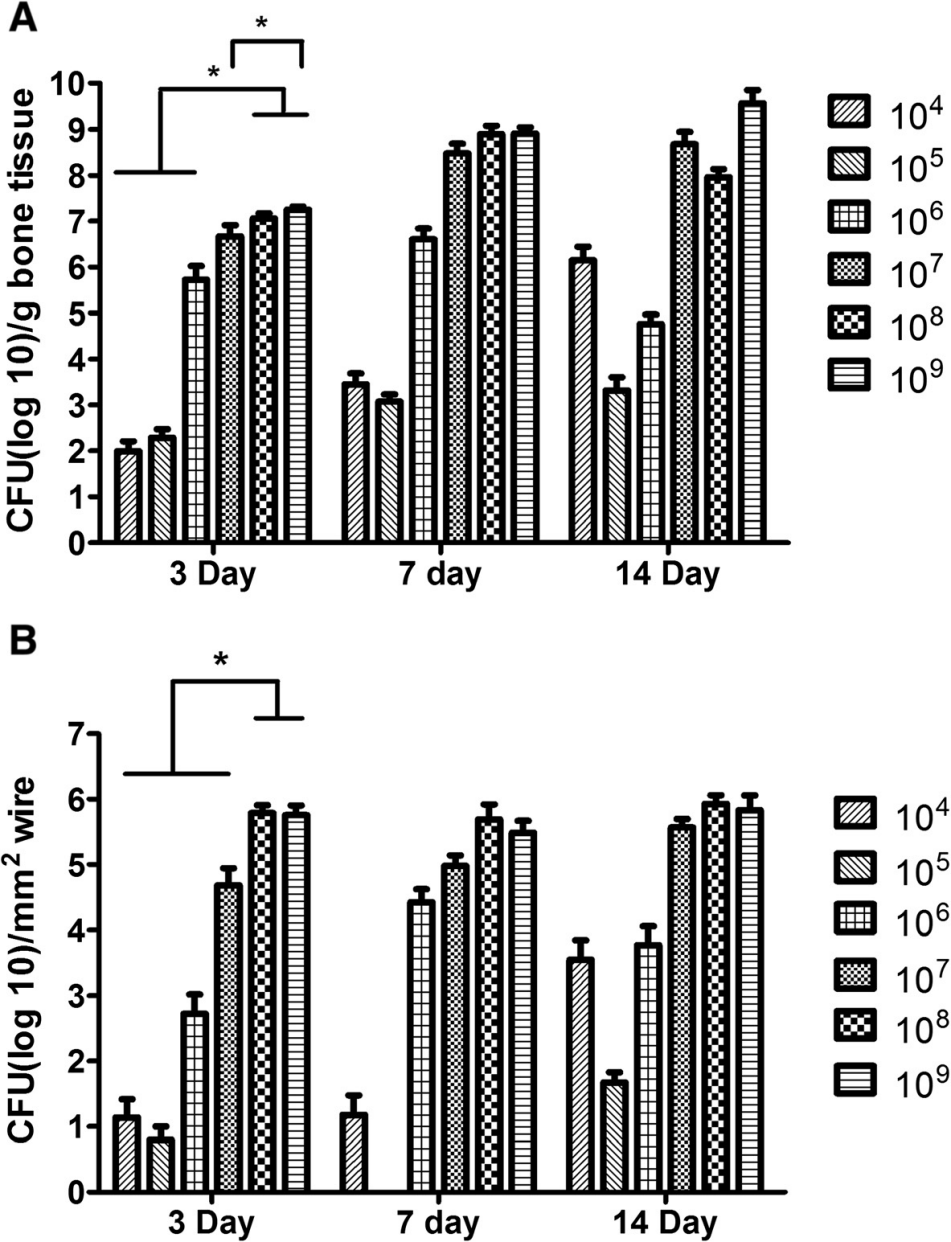
**a** Bacteria colony forming units within the bone. CFU(log10)/g bone tissue and (**b**) Bacteria colony forming units on the K-wire. CFU(log10)/mm^2^ surface area of K-wire. One-way ANOVA performed amongst each inoculum over each time point. Day 3 bone had statistical difference between 10^4^- 10^6^ and 10^8^ & 10^9^. 10^7^ was different than 10^9^ within the bone. Within the wire samples. 10^8^ and 10^9^ groups were significantly greater than the rest of the groups at day 3. There was no difference in the bone or wire at day 7 or 14

Effect of inoculum on infection consistency. The surgery caused the animals in all groups to lose weight initially; the weight of all groups except those inoculated with 10^8^ and 10^9^ had recovered to pre surgery level by day 14 (Fig. 2). There was a dose dependent response between the inoculum size and infection rate (Table 2). It required an inoculum of 10^7^ or higher to establish a reproducible infection (defined as at least 75 % of the samples having ≥10^3^ CFUs). In addition, those inoculated with greater than 10^7^ CFU acquired similar levels of bioburden within the bone and on the wire by day 14 (Fig. 3). Bacteria enumeration within the blood at time of euthanasia demonstrated that no bacteria were detected in groups that received 10^7^ CFU or less. Bacteria was found in the blood of the groups with highest two inoculums (10^8^ and 10^9^), but the levels did not exceed 10^2^ CFU/ml (data not shown); this systemic level may be the reason why the rats in these groups did not gain the weight lost from surgery. Based on the collective data from Experiment 1, we decided to use 10^7^ as the inoculum amount for Experiments 2 and 3.

**Table 2:** Number of positive infected samples in Experiment 1

Inoculum (CFU) Grou		10^4^	10^5^	10^6^	10^7^	10^8^	10^9^
Bone	Day 3	0(2)	0(3)	4(5)	8(8)	7(8)	8(8)
	Day 7	2(8)	2(8)	8(8)	5(6)^a^	8(8)	8(8)
	Day 14	1(1)	1(3)	4(5)	7(8)	8(8)	8(8)
K-Wire	Day 3	0(1)	0(0)	1(1)	5(6)	6(6)	7(7)
	Day 7	0(1)	0(0)	4(4)	5(5)^a^	7(8)	8(8)
	Day 14	1(2)	0(1)	2(3)	7(7)	7(7)	8(8)

Values represent number of positive infected samples with a CFU count ≥ 10^3^ (positive contaminated samples with a CFU count between 10^2^ and 10^3^). All groups out of 8 except ^a^is out of 6

### Experiment 2

**Fig. 4:**
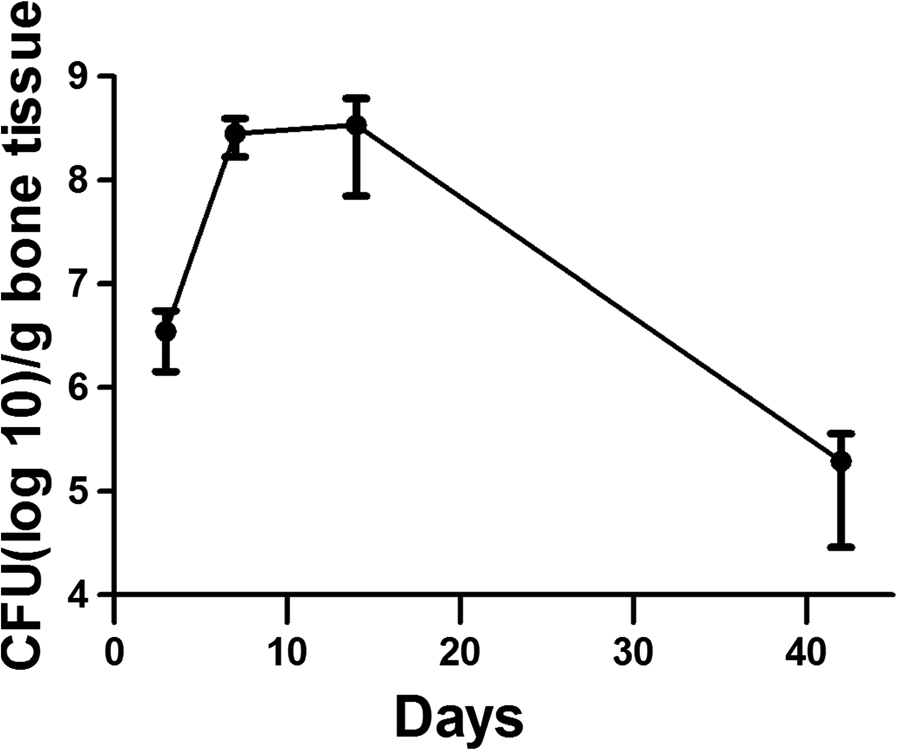
Bacteria CFU in bone when hematogenously inoculated with 10^7^ CFU of *S. aureus*. CFU(log10)/g bone tissue

**Fig. 5:**
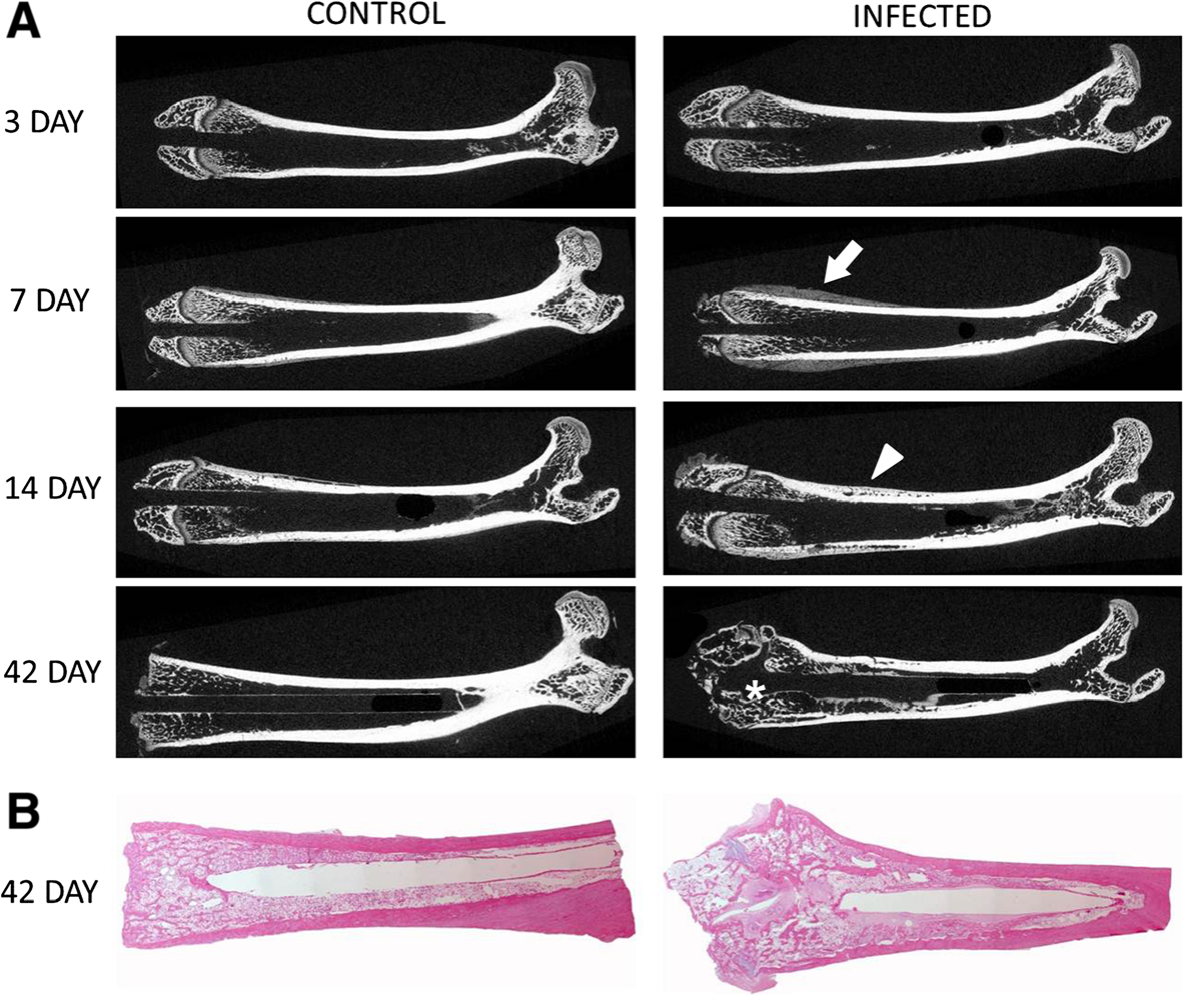
longitudinal μCT sections of femurs at 3, 7, 14, and 42 days for the control (saline) and infected (10^7^ CFU *S. aureus* injected via tail vein) and longitudinal histological sections of bacterial colonization at 42 days post inoculation (stained with H&E) (**a**) progression of morphological changes within the bone between 3 and 42 days post inoculation as seen by μCT. Comparing the control and the infected limbs, the infection is evident by the periosteal reaction seen progressing (*arrow*) forming an involucrum (*arrowhead*). By 42 days, large amounts of boney remodeling are obvious in the infected bone compared to the control (*). **b** 42 days post inoculation with 10^7^ CFU *S. aureus*. The femoral condyle shows signs of bone remodeling

**Fig. 6:**
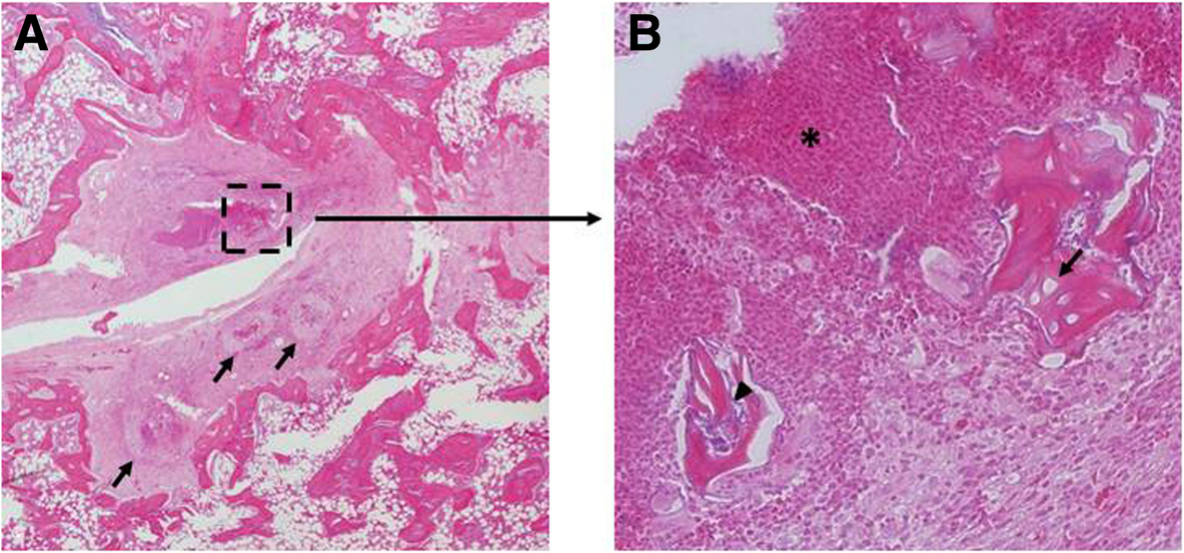
Histological evidence of bacterial colonization. **a** arrows identify loci of colonization with the characteristic fibrous tissue surrounding the infection. **b** Necrotic bone tissue evident by the empty lacunae (arrow), localized bacteria (arrowhead), and agglomeration of neutrophils (*)

Effect of a hematogenous infection on bone morphology. As time progressed, animal weights returned and surpassed pre-operative weights and the infection within the bone reduced but was still as high as 10^5^ CFU/g bone (Fig. 4). There were morphological changes within the bone structure as time progressed (Fig. 5a). By day 7, periosteal reaction is more evident in the infected limbs compared to the control limbs. At day 14, the infection within the femur has caused involucrum and early bone resorption of the cortex along the diaphysis. The greatest change in calcified tissue morphology can be seen at day 42, with large amounts of bone remodeling and resorption. Subjective evaluation of the μCT images reveals a difference in the quality of bone for animals survived for 42 days compared to the controls. Histologically, gross inspection of the sections revealed an increase in cellularity, increase of necrotic tissue (bone marrow) and an increase in hemorrhage of the samples inoculated with *S. aureus* as compared to the control animals that received the surgery but no bacteria at 3 days. There was no obvious difference in neutrophil infiltration between the experimental and control groups at 3 days with similar trauma in the femurs caused by the reaming and insertion of the K-wire. There are signs of increase in necrotic bone marrow at 7 and 14 days as compared to the control groups. At 42 days, however, there is a discernable difference in bone morphology between the control and experimental groups (Fig. 5b). At 42 days, loci of bacterial colonization are evident by the localization of inflammatory cells and the formation of fibrous tissue around necrotic bone tissue, evident by the empty lacunae within the bone fragments. In addition, there is the presence of bacteria within the loci (Fig. 6).

### Experiment 3

**Fig. 7:**
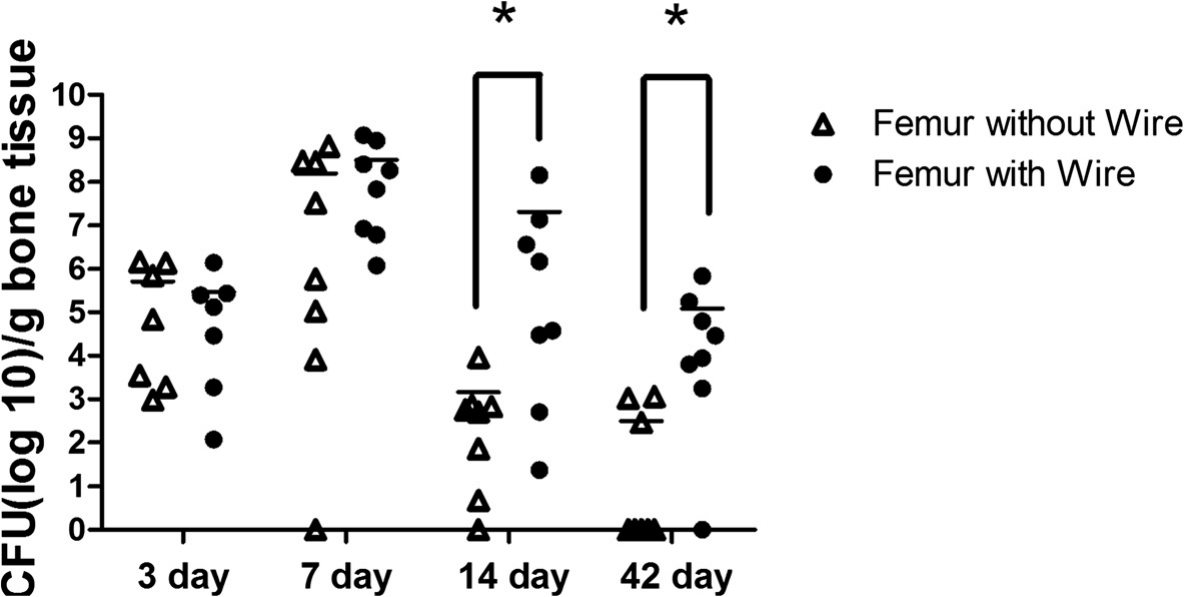
Bacteria colony forming units in bone comparing the bone with an implanted K-wire and the bone with a sham surgery after post-surgical inoculation of 10^7^ CFU *S. aureus* CFU(log10)/g bone tissue. Comparing animals with infection (≥10^3^ CFU) to animals without infection, there is a statistical difference between the implanted limb and the sham limb at 14 and 42 days. Fisher’s exact *p* < 0.05

Effect of an implant on infection rate. It was important to confirm that the addition of the K-wire affected infection rate. It was evident that after 14 days the wire did encourage colonization within the implanted limb (Fig. 7). There was significance (*p* < 0.05; Fisher’s Exact) between the number of limbs infected (≥10^3^ CFU bacteria/g bone) and the number of limbs not colonized (<10^3^ CFU bacteria/g bone) between the wire implanted femur and the sham at day 14 and 42.

## Discussion

Hematogenous orthopedic implant-related infections may develop after prosthetic joint replacement or open-reduction and internal fixation of closed fractures. The risk of an internal fixation device becoming contaminated after a closed fracture is around 0.4 % [7]. The risk of a prosthetic joint becoming infected is between 0.3 and 2.0 %. Although a small percentage, with the total number of total hip arthroplasties in the US 2007 at ~200,000 and total knee arthroplasties around ~550,000, the cost of treating implant-related infections is growing. The small incidence of infection also makes human trials for therapies extremely difficult and costly; therefore, a hematogenous-based implant-related infection model needed. Reizner *et al.* reported no such model in their review of the literature [13]. A rabbit hematogenous implant-related infection model has been attempted, but rabbits are highly susceptible to sepsis from the intravenous bacteria inoculation causing a high mortality rate. Attempts to decrease the bacterial inocula resulted in low and inconsistent infection rate [14–16]. The rat models associated with implant-related osteomyelitis use a direct inoculation method to represent either contamination of an open wound or a surgical site infection. Ozturan *et al.* investigated a chronic osteomyelitis model in which bacteria was injected into the femoral canal prior to placement of a sterile K-wire [17]. Although this model is very useful at evaluating interventions related to implant-related infection, the mode of infection is different than that of a hematogenous infection. To evaluate a material with long-term anti-microbial expectations to prevent osteomyelitis, a more appropriate model would need to be used.

We were able to establish a hematogenously acquired infection on a metallic implant when at least 10^7^ CFU of *S. aureus* was injected via a tail vessel. We saw a dose dependent effect when increasing the number of bacteria injected from 10^4^ to 10^7^, similar to an effect noted by our laboratory when the bacteria was introduced locally [18]. The number of bacteria inoculated, either directly or hematogenously, relates directly to the ability of the bone to become infected. Using 10^7^ CFU, a consistent infection of the implant and femur was established without affecting body weight and without other indications of sepsis. Higher numbers of *S. aureus*, that is 10^8^ and 10^9^ CFU, showed consistent colonization of the implant; however, the animals lost weight after surgery and after 14 days, didn’t regain the weight. Bioburden within the kidneys and spleen was also measured (data not shown) which confirmed that the 10^7^ *S. aureus* inoculation had a smaller systemic effect than the 10^8^ and 10^9^ inoculations. By day 14, animals inoculated with 10^8^ and 10^9^ CFU had 1-4x10^8^ CFU/g kidney tissue compared to those animals inoculated with 10^7^ CFU, which had 4-log lower bacteria in the kidney. It was determined based on the consistency of the infection and the health of the animal, that 10^7^ CFU of this strain of *S. aureus* is relatable to early signs of osteomyelitis that patients may acquire on their metallic orthopedic implants. Presentation of infection depends greatly on the type of surgical implant, that is, fracture fixation or prosthetic devices. Within fixation devices, infections can present in a various of ways depending on a multitude of factors including the type of preceding trauma and the quality of bone [7]. Early onset infections can be characterized by local hyperthermia and prolonged wound healing. Delayed and chronic infections manifest as persistant pain and signs of local inflammation. Prosthetic joint infections can be first noticed as new onset joint pain. If left untreated, the chances of implant loosening increase and implant retention decrease [7]. 10^7^ CFU injected via a tail vein was able to produce a clinically defined infection within the bone with the K-wire after three days, with bioburden measured at 4.6 × 10^6^ CFU/g bone tissue and continue to increase to day 14 at 4.8 × 10^8^ CFU/g bone tissue. Establishing a model that mimics the type of infection needing to be treated is key to continue to investigate treatment strategies.

It was apparent that the incorporation of an implant increased the incidence of an infection after 14 days. It is well known that the presence of a foreign body, such as an implant, increases the risk of infection because the implant acts as a niche for microbial colonization. Gristina *et al.* introduced the concept of the race to the surface, a competition between host and microbial cells [19]. In cases where there will be minimal tissue-implant integration, thus normal immune responses are compromised, the implant is more susceptible to bacteria attachment [19]. Since hematogenous infections are often diagnosed after prolonged periods of implantation, which is accompanied by bone regeneration and scar tissue formation, a model which investigates the rate of infection after prolonged implantation would be more ideal. Our investigation using a time of inoculation at the time of surgery shows that we are able infect an implant via a hematogenous source, however, further investigation into delayed infection is warranted.

Osteomyelitis causes morphological changes in the bone. When not treated, the bone mineral may begin to resorb and the bone tissue may become necrotic. We were able to detect a small change in bone volume via μCT. Also, histology demonstrated inflammation, bone remodeling, and osteonecrosis in the infected femurs. From the histology, we could see a slowly increasing number of inflammatory cells within the marrow space. We also identified areas of osteonecrosis, recognizable by the empty lacunae and areas of sequestra.

## Conclusions

In conclusion, a rat hematogenous osteomyelitis model was characterized as a pre-clinical model for investigating prophylactic and therapeutic treatments for implant-related hematogenous infections. 10^7^ CFU *S. aureus* injected via a tail vein infected the femur and colonized the wire consistently and is an optimal number of bacteria to cause implant-related infections without compromising the overall health of the animal.

## References

[1] Zimmerli W, Trampuz A, Ochsner PE (2004). Prosthetic-joint infections. N Engl J Med.

[2] Johansen LK, Jensen HE (2013). Animal models of hematogenous Staphylococcus aureus osteomyelitis in long bones: a review. Orthop. Res. Rev..

[3] Horst SA, Hoerr V, Beineke A, Kreis C, Tuchscherr L, Kalinka J, Lehne S, Schleicher I, Kohler G, Fuchs T (2012). A novel mouse model of Staphylococcus aureus chronic osteomyelitis that closely mimics the human infection: an integrated view of disease pathogenesis. Am J Pathol.

[4] Hienz SA, Sakamoto H, Flock JI, Morner AC, Reinholt FP, Heimdahl A, Nord CE (1995). Development and characterization of a new model of hematogenous osteomyelitis in the rat. J Infect Dis.

[5] Johansen LK, Frees D, Aalbaek B, Koch J, Iburg T, Nielsen OL, Leifsson PS, Jensen HE (2011). A porcine model of acute, haematogenous, localized osteomyelitis due to Staphylococcus aureus: a pathomorphological study. Acta Pathol. Microbiol. Immunol. Scand..

[6] Scheman L, Janota M, Lewin P (1941). The production of experimental osteomyelitis. JAMA.

[7] Zimmerli W. Clinical presentation and treatment of orthopaedic implant-associated infection. J Intern Med. 2014;276(2):111–9. 10.1111/joim.12233 24605880

[8] Campoccia D, Montanaro L, Arciola CR (2006). The significance of infection related to orthopedic devices and issues of antibiotic resistance. Biomaterials.

[9] Busscher HJ, van der Mei HC, Subbiahdoss G, Jutte PC, van den Dungen JJ, Zaat SA, Schultz MJ, Grainger DW (2012). Biomaterial-associated infection: locating the finish line in the race for the surface. Sci Transl Med.

[10] Chen P, Abercrombie JJ, Jeffrey NR, Leung KP (2012). An improved medium for growing Staphylococcus aureus biofilm. J Microbiol Methods.

[11] Sanchez CJ, Prieto EM, Krueger CA, Zienkiewicz KJ, Romano DR, Ward CL, Akers KS, Guelcher SA, Wenke JC (2013). Effects of local delivery of D-amino acids from biofilm-dispersive scaffolds on infection in contaminated rat segmental defects. Biomaterials.

[12] Penn-Barwell JG, Baker B, Wenke JC. Local bismuth thiols potentiate antibiotics and reduce infection in a contaminated open fracture model. J Orthop Trauma. 2015;29(2):73–8 10.1097/BOT.0000000000000171 24978943

[13] Reizner W, Hunter JG, O'Malley NT, Southgate RD, Schwarz EM, Kates SL (2014). A systematic review of animal models for Staphylococcus aureus osteomyelitis. Eur Cell Mater.

[14] Poultsides LA, Papatheodorou LK, Karachalios TS, Khaldi L, Maniatis A, Petinaki E, Malizos KN (2008). Novel model for studying hematogenous infection in an experimental setting of implant-related infection by a community-acquired methicillin-resistant S. aureus strain. J. Orthop. Res..

[15] Southwood RT, Rice JL, McDonald PJ, Hakendorf PH, Rozenbilds MA (1985). Infection in experimental hip arthroplasties. J. Bone Joint Surg. Br. Vol..

[16] Blomgren G, Lindgren U (1980). The susceptibility of total joint replacement to hematogenous infection in the early postoperative period: an experimental study in the rabbit. Clin Orthop Relat Res.

[17] Ozturan KE, Yucel I, Kocoglu E, Cakici H, Guven M (2010). Efficacy of moxifloxacin compared to teicoplanin in the treatment of implant-related chronic osteomyelitis in rats. J. Orthop. Res..

[18] Penn-Barwell JG, Rand BC, Brown KV, Wenke JC (2014). A versatile model of open-fracture infection: a contaminated segmental rat femur defect. Bone Joint Res.

[19] Gristina A, Naylor P, Myrvik Q (1988). Infections from biomaterials and implants: a race for the surface. Med. Prog. Technol..

